# Novel MASP-2 inhibitors developed via directed evolution of human TFPI1 are potent lectin pathway inhibitors

**DOI:** 10.1074/jbc.RA119.008315

**Published:** 2019-04-05

**Authors:** Dávid Szakács, Andrea Kocsis, Róbert Szász, Péter Gál, Gábor Pál

**Affiliations:** From the ‡ Department of Biochemistry, ELTE Eötvös Loránd University, Pázmány Péter sétány 1/C, H-1117 Budapest,; the § Institute of Enzymology, Research Centre for Natural Sciences, Hungarian Academy of Sciences, Magyar tudósok körútja 2, H-1117 Budapest,; the ¶ Department of Hematology, Institute of Internal Medicine, University of Debrecen, Nagyerdei krt. 98, H-4032 Debrecen, and; ‖ EvolVeritas Biotechnology Ltd., Somogyi Béla u. 17, H-6600 Szentes, Hungary

**Keywords:** innate immunity, complement system, phage display, serine protease, directed evolution, ischemia, protease inhibitor, complement-targeted therapy, MASP-2, lectin pathway, ischemia-reperfusion injury, serine proteinase

## Abstract

The lectin pathway (LP) of the complement system is an important antimicrobial defense mechanism, but it also contributes significantly to ischemia reperfusion injury (IRI) associated with myocardial infarct, stroke, and several other clinical conditions. Mannan-binding lectin–associated serine proteinase 2 (MASP-2) is essential for LP activation, and therefore, it is a potential drug target. We have previously developed the first two generations of MASP-2 inhibitors by *in vitro* evolution of two unrelated canonical serine proteinase inhibitors. These inhibitors were selective LP inhibitors, but their nonhuman origin rendered them suboptimal lead molecules for drug development. Here, we present our third-generation MASP-2 inhibitors that were developed based on a human inhibitor scaffold. We subjected the second Kunitz domain of human tissue factor pathway inhibitor 1 (TFPI1 D2) to directed evolution using phage display to yield inhibitors against human and rat MASP-2. These novel TFPI1-based MASP-2 inhibitor (TFMI-2) variants are potent and selective LP inhibitors in both human and rat serum. Directed evolution of the first Kunitz domain of TFPI1 had already yielded the potent kallikrein inhibitor, Kalbitor® (ecallantide), which is an FDA-approved drug to treat acute attacks of hereditary angioedema. Like hereditary angioedema, acute IRI is also related to the uncontrolled activation of a specific plasma serine proteinase. Therefore, TFMI-2 variants are promising lead molecules for drug development against IRI.

## Introduction

The complement system (CS)^2^ is an essential part of innate immunity. It is a network of more than 30 plasma and cell surface proteins that recognizes, labels, and eliminates microbial pathogens and dangerously altered (*e.g.* apoptotic) self-cells, triggers inflammation, and recruits immune cells (1–3).

The CS can be activated through three pathways. The classical pathway (CP) is activated primarily by immune complexes, but it can also recognize microbial surfaces and apoptotic and necrotic cells; it contributes to the elimination of unnecessary synapses during ontogenesis; and it is important for the clearance of immune complexes and cell debris (4, 5). The lectin pathway (LP) recognizes ancient surface-exposed molecular determinants on microbes via a diverse set of pattern recognition molecules (PRMs) and provides immediate defense against microbial pathogens, which does not depend on specific antibodies (6). The alternative pathway (AP) continuously challenges all surfaces by spontaneous low-level activation, but it activates productively only on those that lack protecting complement regulator molecules. Additionally, the AP provides an important amplification loop for complement activation (7, 8).

Danger signal recognition triggers the activation of pathway-specific serine proteinase zymogens. The activated proteinases cleave downstream complement components that form surface-bound C3 convertases: C4b2a for the CP and the LP and C3bBb for the AP. At this point, the three activation pathways converge to a common effector route leading to the labeling and lysis of the pathogens, recruitment of immune cells, and triggering of inflammation.

Normally, complement activation is tightly regulated (9). Lack of complement inhibition is responsible for the pathomechanism of paroxysmal nocturnal hemoglobinuria, and inappropriate complement activation contributes to the development of diseases such as ischemia-reperfusion injury (IRI), rheumatoid arthritis, age-related macular degeneration, and neurodegenerative diseases including Alzheimer's disease. Therefore, there is a great need for potent and specific anti-complement drugs that could provide targeted therapies for these complement-related diseases (10). Whereas many anti-complement compounds are under development (11), there are only two molecules that have been approved for clinical use: the anti-C5 antibody Soliris® (eculizumab) and C1 inhibitor. Even of these two, only eculizumab is a dedicated complement-targeted drug. One of the most promising candidates among complement-targeted lead molecules is compstatin and its derivatives (12). These compounds effectively block the interaction of the C3 convertases with C3 to inhibit erroneous complement activation.

In most complement-related diseases, the contribution of only one of the three pathways is dominant. The CP participates in Alzheimer's disease (13) and myasthenia gravis (14), the LP contributes to IRI of various tissues (15–18), and the AP plays a significant role in age-related macular degeneration (19) and atypical hemolytic uremic syndrome (20). Pathway-specific inhibitors should be useful tools for academic research to identify individual roles of the three pathways in physiologic and pathologic processes and ideal therapeutics that selectively block the derailed pathologic pathway while leaving the protecting functions of the other two pathways undisturbed.

Pathway-specific proteinases are obvious targets of pathway-selective drug development. However, as most plasma serine proteinases have trypsin-like substrate specificity, selectively targeting a single complement proteinase is a formidable challenge. Small molecules that target only the active site are rarely monospecific. Fragment-based drug discovery (21) has been successfully applied to develop highly selective small-molecule inhibitors against factor D, a key enzyme of the AP (22). These compounds target factor D in its unique, self-inhibited conformation that is characteristically different from other proteinases. Proteins such as mAbs and canonical serine proteinase inhibitors have much larger interacting surfaces and have therefore greater potential to provide monospecificity.

In the last decade we have developed the first selective LP inhibitors by directed evolution of canonical serine proteinase inhibitors (23–25). The first-generation LP inhibitors were based on the 14-amino acid sunflower trypsin inhibitor (SFTI) scaffold, resulting in SFMI-1 and SFMI-2 (23). The second-generation compounds were developed on the scaffold of the 35-amino acid *Schistocerca gregaria* proteinase inhibitor 2 (SGPI-2), yielding SGMI-1 and SGMI-2 (24, 25). With these inhibitors, we revealed that both MASP-1 and MASP-2 are essential for LP activity in human serum (23–25), and therefore, both enzymes are promising targets for drug development. However, MASP-1 has several functions outside the LP (26–29), and MASP-2 has a significantly lower plasma concentration than MASP-1 (30). Therefore, MASP-2 might be a better target for the development of highly selective LP inhibitors.

Nonhuman origin rendered the previously developed MASP inhibitors suboptimal for subsequent drug development. Therefore, we decided to develop a third generation of MASP-2 inhibitors based on a human scaffold to reduce the risk of immunogenicity in humans. We chose the factor Xa–inhibiting second Kunitz domain of tissue factor pathway inhibitor 1 (TFPI1 D2), as it is normally present in the plasma and because it has already been shown to be a low-affinity inhibitor of MASP-2 (31). Here, we present how we developed TFPI1-based MASP-2 inhibitor (TFMI-2) variants that potently inhibit both human and rat MASP-2, enabling their use in proof-of-concept studies in rats.

## Results

### Selection of the TFPI1 D2-phage library for binding to human or rat MASP-2

We followed the same strategy we already applied for developing TFPI1 D2–based MASP-3 inhibitors (32). We randomized the P3–P4′ region (33) of TFPI1 D2 (UniProt ID P10646) except the P2 Cys that forms a structurally indispensable disulfide (Fig. S1). The inhibitor-phage library of 5 × 10^8^ clones was selected for binding to the catalytic fragment of human MASP-2 (hMASP-2cf) or that of rat MASP-2 (rMASP-2cf) in separate experiments. Target-binding clones were identified and sequenced to determine sequence patterns that enable binding to hMASP-2cf or rMASP-2cf. Amino acid and DNA sequences of hMASP-2– and rMASP-2–binding clones are listed in Tables S2 and S3, respectively.

### Scaffold-dependent amino acid preferences of hMASP-2 at the evolved inhibitor positions

**Figure 1.**
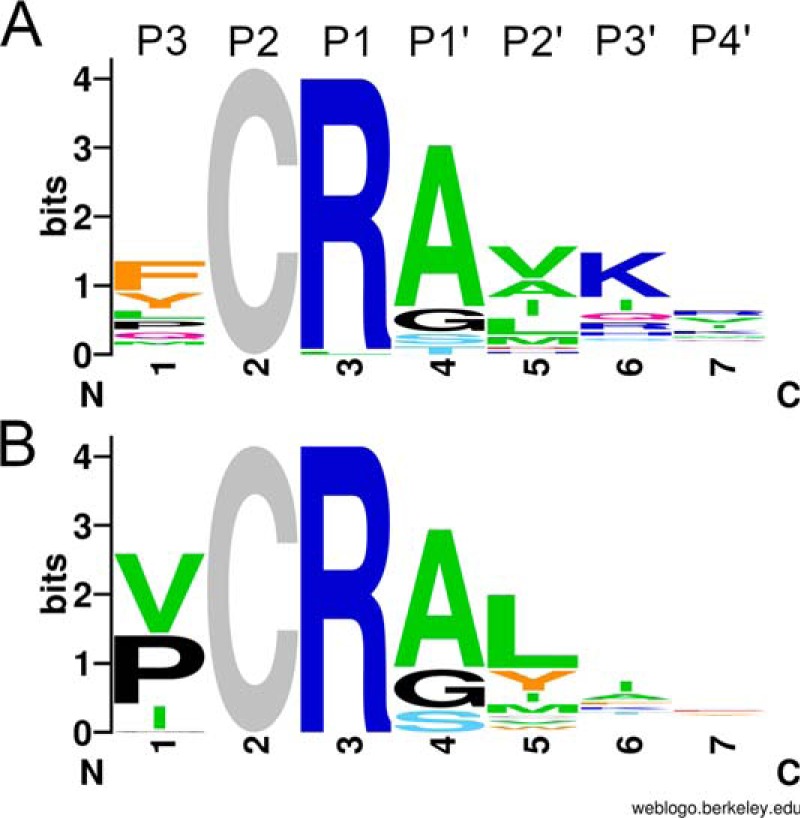
**Codon bias–normalized amino acid frequencies of human and rat MASP-2–selected TFPI1 D2 variants.** *A*, codon bias–normalized sequence logo of human MASP-2 binding clones. *B*, codon bias–normalized sequence logo of rat MASP-2–binding variants. *A* and *B*, positions are labeled according to the Schechter–Berger nomenclature *above* each *column*. The *height* of each *column* represents the degree of conservation. The cysteine at P2 was not randomized; therefore, it shows the *maximal column height. Letter heights* indicate normalized amino acid frequencies. *Colors* reflect the chemical properties of the amino acid side chains.

The codon bias normalized sequence pattern of the hMASP-2–binding TFPI1 D2 clones is presented in Fig. 1*A* as a sequence logo. We compare this logo with those we obtained previously when generating the SFTI-based (23) and the SGPI-based MASP-2 inhibitors (24).

The three unrelated scaffolds stabilize the canonical loop conformation through different intramolecular interactions, including disulfides that we kept intact. Therefore, only the three central loop positions, P1, P1′, and P2′, were randomized on all three scaffolds. Human MASP-2 selected only Lys and/or Arg at the P1 position on all scaffolds, but the relative frequencies of these residues are scaffold-dependent. The enzyme preferred Lys on the SGPI-2 and Arg on the SFTI, whereas it exclusively selected an Arg on the TFPI1 D2 scaffold. Similar scaffold-dependent differences are observed at the P1′ and P2′ positions. At P1′, hMASP-2 preferred Ala/Gly/Ser/Thr on TFPI1 D2, whereas it accepted only Ser/Gly on SFTI and Leu/Ala on SGPI-2. At P2′, on TFPI1 D2 hMASP-2 preferred aliphatic Val/Ala/Ile/Leu, whereas on SGPI-2, it selected only aromatic Trp/Tyr. On SFTI, the preference was a mixture of the previous two, as mostly Tyr/Phe and, to a lesser extent, Ile/Met/Leu were selected. The observed differences between the three selected sequence patterns demonstrate that each unrelated scaffold uniquely affects the side-chain preference of the same enzyme at analogous canonical loop positions.

### hMASP-2 and rMASP-2 share similar amino acid preferences at most evolved TFPI1 D2 positions

Logos derived from the sequences of unique hMASP-2– and rMASP-2–binding TFPI1 D2 clones are shown in Fig. 1, *A* and *B*, respectively. At the two energetically most important positions, the two enzymes selected the same amino acids: Arg at P1 and Ala/Gly/Ser at P1′. At P2′, both enzymes selected hydrophobic side chains; hMASP-2 preferred aliphatic, whereas rMASP-2 both aliphatic and aromatic residues. At P3′ and P4′, hMASP-2 shows a weak preference for positively charged residues, whereas rMASP-2 lacks any observable selectivity.

At P3, there is a clear species-specific difference in amino acid preferences, but even here, there is an overlapping set of selected residues. Whereas rMASP-2 selected only small hydrophobic residues, Val/Pro/Ile/Gly, hMASP-2 preferred Phe/Tyr and selected smaller residues, such as Pro/Val, at lower frequencies.

### Novel MASP-2 inhibitor variants designed based on the sequence logos

We designed three TFMI-2 variants along the notion that normalized amino acid frequencies generally correlate with binding energy contributions of individual amino acid residues (34–37). TFMI-2a carries the hMASP-2–selected consensus P3-P4′ sequence (FCRAVKR) and is expected to have the highest affinity toward hMASP-2. On the other hand, due to its bulky P3 Phe, it should be only a weak inhibitor of rMASP-2. Therefore, we designed two additional inhibitors to efficiently inhibit both hMASP-2 and rMASP-2. Such variants can serve as surrogate compounds in studies investigating the *in vivo* effects of MASP-2 inhibition in rats. As rMASP-2 mostly preferred a Pro or Val at P3, we substituted the P3 Phe with Pro in TFMI-2b (PCRAVKR) and Val in TFMI-2c (VCRAVKR).

### TFMI-2 variants are efficient inhibitors of hMASP-2 and rMASP-2, whereas they are completely inactive against hMASP-1 and hMASP-3

TFPI1 D2 and TFMI-2a-c were expressed in *Escherichia coli* and purified to homogeneity. Their equilibrium inhibitory constants (*K_I_*) were determined against the catalytic fragments of human MASP-1, human and rat MASP-2, and human MASP-3 (Table 1).

**Table 1:** ***K_I_* values of TFPI1 D2, TFMI-2a–c variants, and SGMI-2 on the catalytic fragments of human MASP-1, -2, and -3 and rat MASP-2**

Inhibitor	Sequence (P4–P4′)	*K_I_*			
		Human MASP-1cf	Human MASP-2cf	Rat MASP-2cf	Human MASP-3cf
		*nm*			
TFMI-2a	GFCRAVKR	NE*^a^*	2.0 ± 0.1*^b^*	640 ± 20*^b^*	NE
TFMI-2b	GPCRAVKR	NE	7.9 ± 0.3*^b^*	7.2 ± 0.2*^c^*	30,000*^d^*
TFMI-2c	GVCRAVKR	NE	36.7 ± 0.7*^b^*	7.5 ± 0.3*^b^*	NE
TFPI1 D2	GICRGYIT	NE	1883 ± 48*^b^*	185 ± 4*^b^*	NE
SGMI-2	VCTKLWCN	NE	6*^e^*	22.7 ± 1.6*^c^*	5200 ± 300*^f^*

*^a^* NE, not effective; no inhibition could be detected.

*^b^* Average ± range (*n* = 2).

*^c^* Average ± S.D. (*n* = 3).

*^d^* Approximation based on a single measurement.

*^e^* Data from Ref. 24.

*^f^* Data from Ref. 25.

TFPI1 D2 was previously shown to inhibit hMASP-2 with low affinity (31). We found that TFPI1 D2 is indeed a weak inhibitor of hMASP-2 (*K_I_* = 1883 nm) and a moderate inhibitor of rMASP-2 (*K_I_* = 185 nm).

All TFMI-2 variants are potent, low nanomolar inhibitors of hMASP-2 with 50–940-fold higher affinities toward the enzyme than TFPI1 D2. As expected, TFMI-2a has the highest affinity (*K_I_* = 2.0 nm), and it is 3-fold more potent than SGMI-2, our previous best compound (24). Substituting P3 Phe with Pro in TFMI-2b resulted in a 7.9 nm *K_I_* (4-fold affinity reduction), whereas the introduction of Val at P3 in TFMI-2c resulted in a 36.7 nm *K_I_* value (18-fold affinity drop). Importantly, TFPI1 D2 and the TFMI-2 variants have either no or a negligible inhibitory effect on hMASP-1 and hMASP-3.

In accordance with the sequence logos, TFMI-2b and TFMI-2c are almost equally potent rMASP-2 inhibitors with *K_I_* values of 7.2 and 7.5 nm, respectively. These values represent a 25-fold affinity improvement compared with TFPI1 D2. On the other hand, TFMI-2a is significantly less potent with a *K_I_* value of 640 nm. These results show that the hMASP-2–binding consensus P1–P4′ region (RAVKR) shared in all three TFMI-2 variants is compatible with rMASP-2 inhibition, but as expected, P3 Phe of TFMI-2a is detrimental for binding to the enzyme, causing an almost 100-fold affinity drop. SGMI-2 inhibits rMASP-2 with a *K_I_* value of 22.7 nm.

In all, TFMI-2b and TFMI-2c are 3-fold stronger rMASP-2 inhibitors than SGMI-2, and most importantly, TFMI-2b is an equally potent inhibitor of hMASP-2 and rMASP-2. Based on these *in vitro* data, TFMI-2b can be a suitable surrogate of TFMI-2a in subsequent proof-of-concept studies in rats.

### TFMI-2 variants are potent and selective inhibitors of the LP in normal human serum (NHS)

We characterized the effects of the TFMI-2 variants on LP activation in several complement assays and compared the half-maximal inhibitory concentration (IC_50_) values with those of SGMI-2.

### TFMI-2 variants are selective inhibitors of the LP according to the Wieslab test

**Figure 2.**
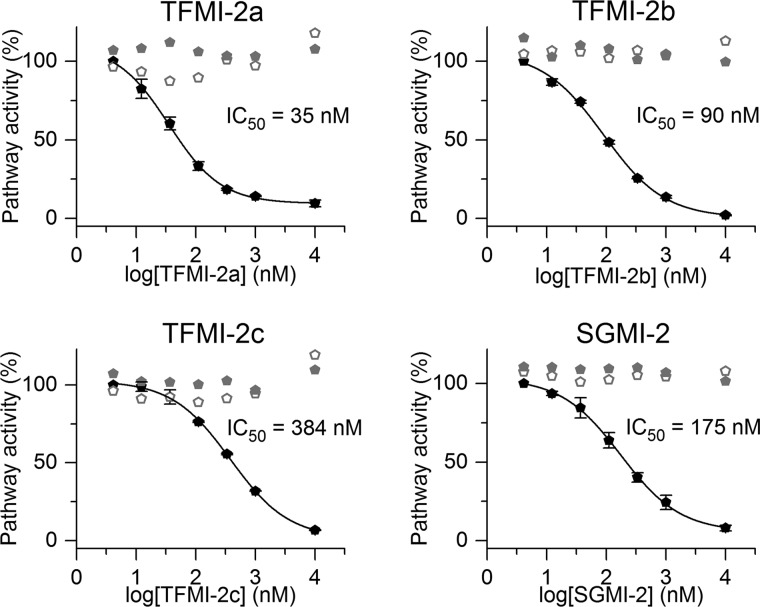
**Effects of the TFMI-2 variants and SGMI-2 were selectively determined on the three individual complement pathways by using the Wieslab kit.** All inhibitors inhibited the lectin pathway (*black pentagons*) in a concentration-dependent manner, whereas the activities of the classical (*open gray pentagons*) and the alternative (*solid gray pentagons*) pathways were unaffected. The IC_50_ values are between 35 and 384 nm (Table 2). TFMI-2a and TFMI-2b are more efficient than SGMI-2, whereas TFMI-2c is the least efficient LP inhibitor in the set. Data points represent the average of two experiments, whereas the *error bars* represent the S.E.

Wieslab experiments demonstrated that the TFMI-2 variants are potent LP inhibitors, with IC_50_ values of 35–384 nm (Fig. 2 and Table 2), whereas none of them inhibit the CP and the AP. Compared with SGMI-2, TFMI-2a and TFMI-2b were more whereas TFMI-2c was less efficient.

**Table 2:**
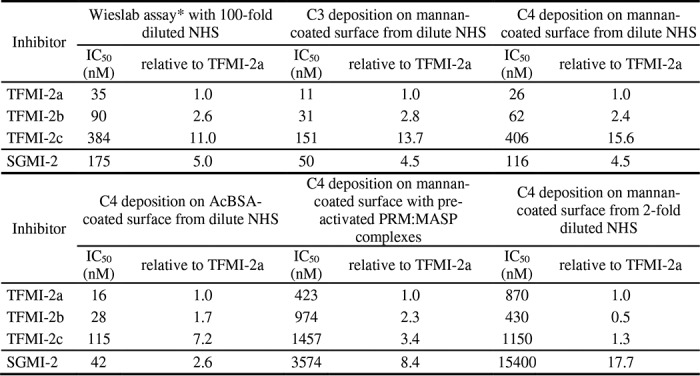
**IC_50_ values of the TFMI-2 variants and SGMI-2 in various complement ELISA tests** For easier comparison, values normalized to those of TFMI-2a are also listed. *, with the Wieslab (previously known as WiELISA) kit (67), one can measure the activation of the three CS pathways independently. In this table, only the results of the LP-selective assays are shown.

### LP-inhibitory potencies of the TFMI-2 variants were characterized in various C3 and C4 deposition assays using diluted NHS

**Figure 3.**
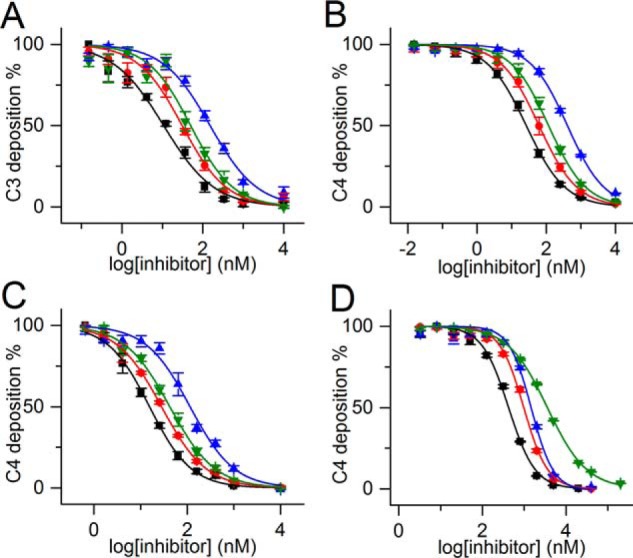
**LP-inhibitory effects of TFMI-2 variants and SGMI-2 in various complement deposition ELISA tests with NHS.** Shown are LP-inhibitory effects of TFMI-2a (■), TFMI-2b (●), TFMI-2c (▴), and SGMI-2 (▾) on C3 deposition ELISA on a mannan-coated surface with 100-fold diluted NHS (*A*), C4 deposition ELISA on a mannan-coated surface with 60-fold diluted NHS (*B*), C4 deposition ELISA on an AcBSA-coated surface with 60-fold diluted NHS (*C*), and deposition of the C4b fragment of purified C4 by pre-activated PRM:MASP complexes generated on a mannan-coated surface (*D*). *Data points* represent the average of four experiments, whereas the *error bars* represent the S.E. The corresponding IC_50_ values are listed in Table 2.

In ELISA tests using diluted NHS, we measured both C3 and C4 deposition on mannan-coated and C4 deposition on acetylated BSA (AcBSA)-coated surfaces. We found that TFMI-2 variants are efficient LP inhibitors both in MBL-dependent and ficolin-dependent assays (Fig. 3, *A–C* and Table 2) with IC_50_ values in the 11–406 nm range. Just as in the Wieslab assay, TFMI-2a and TFMI-2b were more whereas TFMI-2c was less potent than SGMI-2 in these tests.

### TFMI-2 variants inhibit C4 deposition driven by pre-activated PRM:MASP complexes

In the above mentioned assays, the inhibitors were co-incubated with the serum prior to transferring the samples on the activator surface. In contrast, in the first phase of this assay, we generated activated, surface-bound PRM:MASP complexes by incubating 2-fold diluted NHS on mannan-coated surface at high ionic strength. Under these conditions, PRMs attach to the surface and the MASPs activate, but deposition of C4 fragments is prevented (23, 38, 39). After washing out the unbound components, we added purified C4, pre-incubated with the inhibitors in a physiological buffer. The IC_50_ values obtained for SGMI-2 and the three TFMI-2s were between 423 and 3574 nm (Fig. 3*D* and Table 2).

Importantly, all inhibitors, if applied at a high enough concentration, could completely block C4 deposition even without pre-incubation with MASP-2, demonstrating that these compounds rapidly form stable ternary PRM:MASP-2:inhibitor complexes. Interestingly, we found that in this assay, all TFMI-2 variants were superior to SGMI-2.

### TFMI-2 variants are LP-selective inhibitors in 2-fold diluted NHS and are significantly more potent than SGMI-2

**Figure 4.**
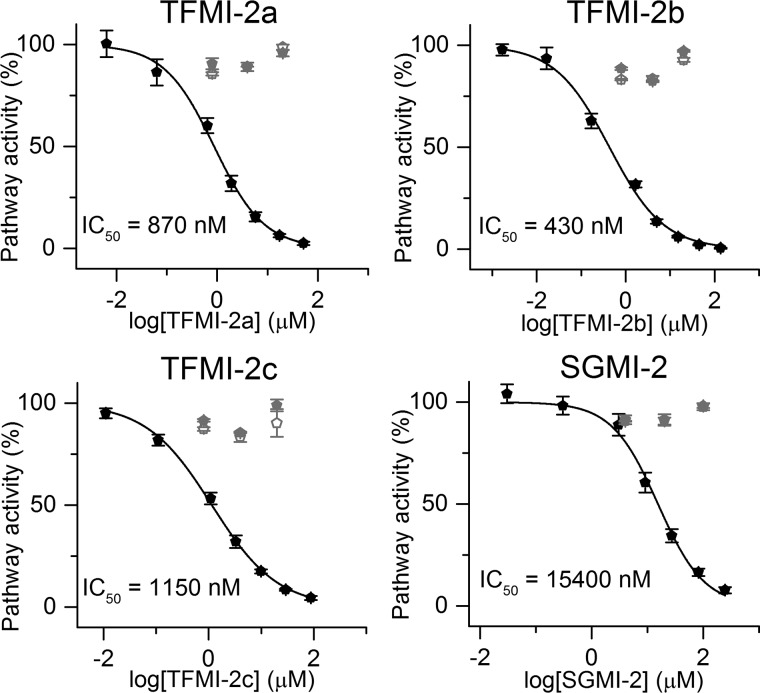
**Inhibitory effects of TFMI-2 variants and SGMI-2 in 2-fold diluted NHS assessed by pathway-selective ELISA tests.** All inhibitors inhibited the lectin pathway (*black pentagons*) in a concentration-dependent manner, whereas the activities of the classical (*open gray pentagons*) and the alternative (*solid gray pentagons*) pathways were unaffected. The IC_50_ values are between 430 nm and 15.4 μm (Table 2). In terms of IC_50_ value ratios, all TFMI-2 variants are 13.4–35.8-fold more potent LP inhibitors than SGMI-2. Data points represent the average of four (in the case of the LP) or two (in the case of the CP and the AP) experiments. The *error bars* represent the S.E.

We also performed pathway-selective ELISAs in more concentrated, 2-fold diluted NHS (25) (Fig. 4 and Table 2). In the LP assays, we used 100 μg/ml sodium polyanethole sulfonate (SPS) to selectively suppress the activation of the CP and the AP (40, 41) and detected C4 deposition. In this assay, all TFMI-2 variants were significantly, 13.4–35.8-fold more potent than SGMI-2 (Fig. 4 and Table 2). Interestingly, in this assay, TFMI-2b was the most potent LP inhibitor, with 2-fold lower IC_50_ than the second best TFMI-2a (Table 2).

In the SPS-free CP and AP assays, we applied inhibitor concentrations that provided up to 90% inhibition in the LP assays. In the CP assay, the surface was coated with human IgG, and deposited C4 was detected. In the AP assay, we prevented the activation of the CP and the LP by using an EGTA-containing buffer free from Ca^2+^ ions and measured C3 deposition. The TFMI-2 variants were completely inactive in these CP and AP assays, demonstrating that TFMI-2s are efficient and LP-specific inhibitors even at high serum concentration (Fig. 4).

### TFMI-2b inhibits the LP in rat serum

**Figure 5.**
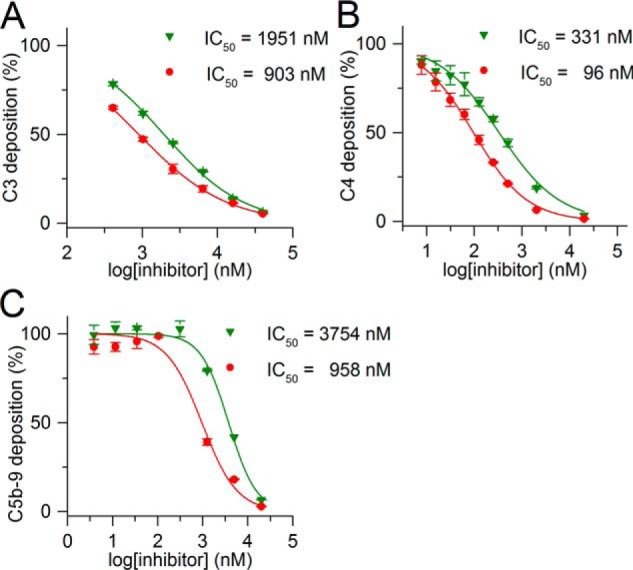
**LP-inhibitory effects of TFMI-2b and SGMI-2 in complement deposition ELISA with rat serum.** Shown are LP-inhibitory effects of TFMI-2b (●) and SGMI-2 (▾) in C3 deposition ELISA on a mannan-coated surface with 70-fold diluted rat serum (*A*), C4 deposition ELISA on a mannan-coated surface with 60-fold diluted rat serum (*B*), and C5b-9 deposition ELISA on a mannan-coated surface with 50-fold diluted rat serum (*C*). Data points represent the average of two experiments, whereas the *error bars* represent the S.E.

We performed LP ELISA tests on mannan-coated plates with diluted individual sera of Wistar rats. We detected the deposition of C3, C4, or the terminal complement antigen C5b-9 in three different assays and compared the inhibitory potencies of TFMI-2b and SGMI-2 (Fig. 5 and Table 3). TFMI-2b proved to be more efficient than SGMI-2 in all three assays with IC_50_ values 2.2–3.9-fold lower than those of SGMI-2 (Table 3). The IC_50_ ratios of the two inhibitors are in agreement with their corresponding *K_I_* ratios on rMASP-2 (Table 1). These data demonstrate that TFMI-2b is active in rat serum, which is a prerequisite for using it as a surrogate of TFMI-2a in *in vivo* rat studies.

**Table 3:** **IC_50_ values of TFMI-2b and SGMI-2 in complement ELISA tests with rat serum** For easier comparison, values normalized to those of TFMI-2b are also listed.

Inhibitor	C3 deposition		C4 deposition		C5b-9 deposition	
	IC_50_	Relative to TFMI-2b	IC_50_	Relative to TFMI-2b	IC_50_	Relative to TFMI-2b
	*nm*		*nm*		*nm*	
TFMI-2b	903	1	96	1	958	1
SGMI-2	1951	2.2	331	3.4	3754	3.9

### TFMI-2 variants do not inhibit blood coagulation

**Figure 6.**
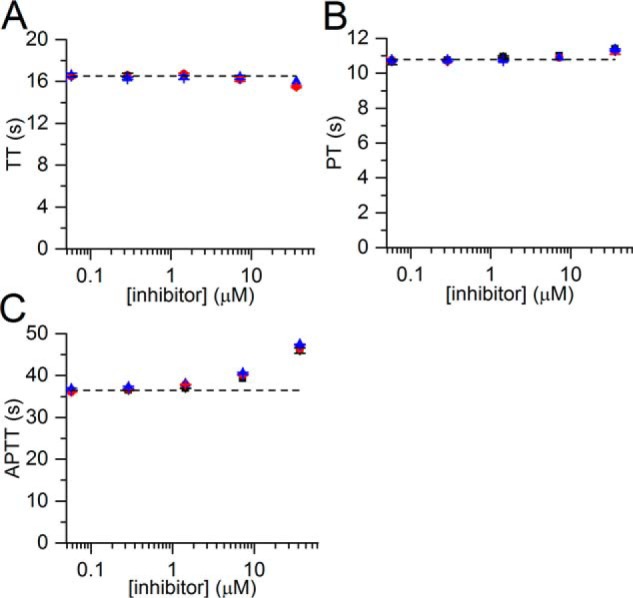
**Effects of the TFMI-2 variants in the three standard blood coagulation tests.** Shown are the effects of TFMI-2a (■), TFMI-2b (●), and TFMI-2c (▴) in the three standard blood coagulation tests: TT (*A*), PT (*B*), and APTT (*C*). The inhibitors do not affect the TT and the PT values and cause a minor increase in the APTT when applied in high concentrations. All *data points* represent the average of two measurements. *Error bars*, S.E. Control values were averaged from six measurements with blood containing no inhibitors and are represented as a *dashed line*.

We tested whether the TFMI-2 variants interfere with the coagulation process in three standard assays, the thrombin time (TT), the prothrombin time (PT), and the activated partial thromboplastin time (APTT). The inhibitors were applied in a 5-fold serial dilution, reaching a highest final concentration of 36 μm, which is 3–4 orders of magnitude higher than the *K_I_* values of the inhibitors toward hMASP-2. Even at the highest concentration, TFMI-2 variants have no effect in the PT and TT tests (Fig. 6, *A* and *B*) and have only a negligible effect in the APTT test (Fig. 6*C*).

## Discussion

Complement LP is a powerful antimicrobial mechanism of innate immunity that is important for maintaining immune homeostasis. However, inappropriate or uncontrolled LP activation contributes to several types of IRI. In mice, genetic MASP-2 deficiency or pretreatment with MASP-2–inhibitory mAbs was shown to result in smaller infarction size in myocardial and gastrointestinal IR injury models (16, 42) and to reduce neurological deficit and histopathological lesion after focal cerebral ischemia (18). MAp44 (MAP-1) is an endogenous LP inhibitor, which binds to the PRMs of the LP and competes off the MASP enzymes. MAp44 was effective in attenuating myocardial IRI (43), and an antibody against MBL, a major PRM of the LP, provided similar effects (44).

In humans, MBL deficiency is associated with a smaller infarction size and favorable outcome after ischemic stroke (45). Moreover, consumption of ficolin-2 (46) and activation of MASP-2, both suggesting LP contribution, were also observed in myocardial IRI (47). A comprehensive recent summary of the field is available in the review of Panagiotou *et al.* (48). Although many smaller studies convincingly associated human IRI with uncontrolled LP activation, data from IRI-related clinical trials on selective LP inhibitors, involving a much larger patient cohort, are still being awaited.

In various studies, Schwaeble and co-workers (16, 18) demonstrated that MASP-2 deficiency or MASP-2 depletion provides significant protection against experimental IRI in cardiac, brain, and gastrointestinal tissues. In these experiments, the authors used MASP-2 KO mice or MASP-2–specific ablating antibodies. The administration of the mAbs always preceded the experimentally induced ischemia by hours or even days (16, 18). From a therapeutic aspect, the requirement for such a long pretreatment with a drug is compatible only with predictable cases of IRI accompanying organ transplantation and other scheduled surgeries or with chronic diseases where sustained MASP-2 inhibition is needed. Interestingly, all ongoing clinical trials related to anti-MASP-2 mAb (OMS721) initiated by OMEROS target chronic diseases such as thrombotic microangiopathies (NCT02222545), IgA nephropathy, lupus nephritis, membranous nephropathy and C3 glomerulopathy (NCT02682407), or atypical hemolytic uremic syndrome (NCT03205995), whereas targeting acute clinical conditions, such as IRI accompanying myocardial infarct or stroke, have not been reported yet. In this paper, we presented our third-generation small-protein inhibitors, the TFMI-2s that have been developed via directed evolution of human TFPI1 D2 to minimize the risk of immunogenicity and thereby maximize therapeutic potential.

In terms of fundamental research, these third-generation MASP-2 inhibitors provided additional data on how canonical serine proteinase inhibitors work. Over 20 years ago, Laskowski and co-workers concluded that the sequence of the canonical binding loop autonomously determines the specificity and affinity of serine protease inhibitors (49) (*i.e.* the role of the scaffold is indirect (50)). They named this phenomenon interscaffolding additivity.

Our first two generations of MASP-2 inhibitors already revealed that inhibitory loop sequences of canonical serine proteinase inhibitors evolved against the same enzyme can be characteristically scaffold-dependent (23–25). The unrelated third-generation MASP-2 inhibitors provided additional evidence that the sequence of the canonical binding loop does not autonomously determine specificity and affinity, verifying that interscaffolding additivity is not a general phenomenon.

In a very recent study, we used pancreatic digestive proteases, trypsin and chymotrypsin, and two unrelated inhibitors and demonstrated that the canonical loop does not act independently of the scaffold (37). Instead, the loop and the scaffold constitute one inseparable functional unit, and different parts of the molecules need to be coevolved to provide stable and highly functional inhibitors (37). Although directed evolution via phage display is a powerful tool to develop serine proteinase inhibitors with novel specificities (51), grafting an optimized inhibitor loop onto an unrelated scaffold is unlikely to be successful (37). These observations should provide valuable information for all those who aim at developing novel proteinase inhibitors.

In terms of translational research, our third-generation inhibitors have important promising qualities as follows. All tested TFMI-2 variants are selective MASP-2 inhibitors and are inactive against MASP-1 and MASP-3. They inhibit only the LP while leaving the CP, AP, and the common pathway of the CS perfectly intact. This means that TFMI-2 variants do not inhibit C1s, C1r, factor D, the C3-convertases C4b2a, C3bBb, and their related C5-convertases. Demonstrated pathway specificity of the TFMI-2s would ensure that whereas LP activation is temporarily shut down for therapeutic purpose, the other two complement pathways would still provide their vital functions.

Importantly, TFMI-2s completely and instantaneously inhibit C4 deposition via already surface-deposited and pre-activated PRM:MASP complexes, demonstrating that TFMI-2s can readily form ternary PRM:MASP-2:inhibitor complexes. This feature should be important for the treatment of acute IRI (*e.g.* in myocardial infarct and stroke).

TFMI-2a carries a bulky Phe at the P3 position, which is optimal for hMASP-2 inhibition but deleterious for rMASP-2 binding. Therefore, TFMI-2a is unsuitable for proof-of-concept studies in rats. As we anticipated such species-specific incompatibilities, we designed our set of third-generation MASP-2 inhibitors to contain at least some variants that are equally potent against human and rat MASP-2, to enable subsequent proof-of-concept studies. Indeed, this approach yielded variants, including TFMI-2b, that could serve as surrogates of TFMI-2a. A suitable surrogate molecule should resemble the clinical candidate as much as possible with regard to production process, impurity profile, affinity, and pharmacological mechanism (52, 53). TFMI-2b, which is a single-point mutant of TFMI-2a, meets these requirements and outperforms SGMI-2 in all LP-inhibitory tests, both in human and rat serum.

TFPI1, harboring TFPI1 D2, the parent molecule of our TFMI-2s, is present in the human plasma and is an important regulator of coagulation. TFPI1 is a potent natural fXa inhibitor (54) via TFPI1 D2 (55, 56) and inhibits coagulation assessed by the PT and APTT tests (54). Importantly, according to the results of our PT, TT, and APTT tests, TFMI-2s do not inhibit any of the six coagulation serine proteinases: thrombin, fVIIa, fIXa, fXa, fXIa, and fXIIa.

The first Kunitz domain of TFPI1 has already been utilized in a phage display–based drug development project that yielded the potent plasma kallikrein inhibitor, ecallantide (Kalbitor®), which is an FDA-approved drug for the treatment of acute attacks of hereditary angioedema (57, 58). This demonstrates that *in vitro* evolved Kunitz domain derivatives of TFPI1 are suitable for human therapy. We chose the second Kunitz domain of human TFPI1 (TFPI1 D2) as the starting molecule, as it had previously been shown to weakly inhibit MASP-2 (31). The IC_50_ of recombinant TFPI1 for LP inhibition, even in 100-fold diluted NHS, was determined to be 10 μm (31). At the reported 2.25 nm plasma concentration of TFPI1 (59), this low inhibitory effect is physiologically irrelevant. On the other hand, the ability of TFPI1 D2 to weakly interact with MASP-2 indicated for us that the molecule could be evolved into an efficient MASP-2 inhibitor, which turned to be the case.

Ecallantide (Kalbitor®) shares considerable similarity with the TFMI-2 variants, and it is repeatedly used to alleviate potentially life-threatening angioedema attacks in HAE patients. Moreover, its half-life was successfully increased by PEGylation.

This suggests that our TFPI D2–based MASP-2 inhibitors could be suitable for acute treatment of life-threatening disease conditions accompanied by IRI, such as myocardial infarct or stroke. Moreover, their serum half-lives could also be optimized by standard methods to enable their use in chronic diseases and predictable cases of IRI. Animal studies with the compounds to assess pharmacokinetics, pharmacodynamics, and *in vivo* efficacy are under way.

## Experimental procedures

### Recombinant MASP fragments

The three-domain catalytic fragments of hMASP-1, hMASP-2, and hMASP-3 were produced as described previously (60–62). These fragments are catalytically equivalent to the full-length enzymes (61, 63). rMASP-2cf was produced similarly to hMASP-2cf (61). rMASP-2cf starts with Gln-298 and ends with Phe-685 according to the sequence of UniProt entry Q9JJS8 and was produced with an extra Met-Thr dipeptide at the N terminus.

### Construction of the TFPI1 D2 library

The TFPI1 D2 library was identical to the one published previously (32). The phagemid vector encodes a fusion protein consisting of an N-terminal FLAG tag, TFPI1 D2 and the p8 coat protein, connected by Ser/Gly linkers (Fig. S1). The sequences of the mutagenesis primers are listed in Table S1.

### Selection and identification of hMASP-2– and rMASP-2–binding variants

Human and rat MASP-2cf (20 μg/ml) were immobilized on MaxiSorp (Nunc) plates in 200 mm sodium carbonate, pH 9.4, at room temperature for 2 h. Three selection and amplification cycles were performed, and individual clones from the second and third selection cycles were tested for target binding. Sequences of 43 unique hMASP-2–binding (Table S2) and 53 unique rMASP-2–binding (Table S3) clones were determined.

### Sequence logo generation

Amino acid frequencies were normalized to the expected initial codon frequencies in the NNK codon set to eliminate codon bias as described (36), and the corresponding sequence logo was created by the WebLogo program (64).

### Construction, expression, and purification of TFPI1 D2 and the TFMI-2 variants

Three TFPI1 D2–based MASP-2 inhibitor (TFMI-2) variants were designed. The DNA fragment encoding TFPI1 D2 was cloned into a modified pMal p2G phagemid vector. This vector was used as a template to produce the TFMI-2a-encoding DNA by Kunkel mutagenesis (65). The DNA fragments encoding TFMI-2b and TFMI-2c were produced by megaprimer mutagenesis. All inhibitor genes were cloned into the S100A4 fusion expression vector described previously (32). The sequence of the gene and the encoded fusion protein is shown in Fig. S2. Sequences of the oligonucleotides are shown in Table S1.

The inhibitors were expressed and purified as described previously (32). Correct molecular mass values of the inhibitors were confirmed by electrospray ionization-MS. The concentration of the inhibitor variants was determined by titration against active site–titrated trypsin.

### Determination of the equilibrium inhibitory constants on the MASP enzymes

The experiments were performed as desribed previously (23), with some modifications. The *K_I_* values of TFPI1 D2 and the TFMI-2 variants on human and rat MASP-2cf, hMASP-1cf, and hMASP-3cf and the *K_I_* value of SGMI-2 on rMASP-2cf were determined in a 200 μl final assay volume in 20 mm HEPES, 145 mm NaCl, 5 mm CaCl_2_, 0.05% Triton X-100, pH 7.5, buffer on 96-well microtiter plates using a BioTek Synergy H4 microplate reader. Constant concentrations of the enzymes were co-incubated with serial dilutions of the inhibitors for 2 h at room temperature. 250 μm Z-Lys-SBzl thioester substrate and 500 μm 5,5′-dithiobis(2-nitrobenzoic acid) co-substrate were added, and the residual enzyme activity was measured at 410 nm in at least two parallel experiments.

The *K_I_* values were determined using the OriginPro software based on the following equation,

(Eq. 1) $$\left[E\right]={\left[E\right]}_{0}-\frac{{\left[E\right]}_{0}+{\left[I\right]}_{0}+K_{I}-\sqrt{{\left({\left[E\right]}_{0}+{\left[I\right]}_{0}+K_{I}\right)}^{2}-4{\left[E\right]}_{0}{\left[I\right]}_{0}}}{2}$$

where [*E*], [*E*]_0_, and [*I*]_0_ represent the molar concentration of the free enzyme, the total enzyme, and total inhibitor, respectively.

### Complement ELISAs with NHS

All assays were performed using NHS pooled from at least 10 healthy individuals. Photometric signals were recorded using a PerkinElmer EnSpire microplate reader. IC_50_ values were calculated using the OriginPro software. Residual complement activities were plotted as the function of the logarithm (log_10_) of the inhibitor concentration, and the DoseResp function was fitted to the data.

### Wieslab tests

To separately test the effect of the TFMI-2s on the three complement pathways, Wieslab COMPL 300 tests were performed according to the manufacturer's protocol with some modifications (23). Two parallels were measured for each data point.

### C3 deposition ELISAs with diluted NHS

C3 deposition was measured based on the work of Møller-Kristensen *et al.* 66 and Kocsis *et al.* (23) with some modifications. Greiner high-binding microtiter plates were coated with 10 μg/ml mannan dissolved in 50 mm sodium carbonate, pH 9.6, buffer (coating buffer). Wells were blocked for 1 h with 1% BSA, 50 mm Tris, 150 mm NaCl, 0.1% Tween 20, pH 7.4, buffer (TBS/BSA/T) and washed with 50 mm Tris, 150 mm NaCl, 5 mm CaCl_2_, 0.1% Tween 20, pH 7.4, buffer (TBS/Ca/T). NHS was diluted 100-fold in 10 mm HEPES, 150 mm NaCl, 5 mm CaCl_2_, 5 mm MgCl_2_, 0.1% Tween 20, pH 7.4, buffer (serum dilution buffer) and incubated with serial dilutions of the inhibitors for 30 min at room temperature. Samples were transferred onto the plate and incubated for 30 min at 37 °C. The plate was rinsed with TBS/Ca/T, followed by applying polyclonal rabbit anti-human C3c antibody (A0062, DakoCytomation) diluted 2,000-fold in TBS/BSA/Ca/T to the plate, which was incubated for 1 h at 37 °C. After washing, horseradish peroxidase–conjugated monoclonal mouse anti-rabbit IgG antibody (A1949, Sigma-Aldrich) diluted 40,000-fold in TBS/BSA/Ca/T was added to the wells, and the plate was incubated for 30 min at 37 °C. The plate was washed, and 1 mg/ml *o*-phenylenediamine dihydrochloride chromogenic peroxidase substrate in 50 mm citrate, 0.1% H_2_O_2_, pH 5.0, buffer was added. After signal development (3–5 min), the reaction was stopped by 50 μl/well of 1 m H_2_SO_4_, and the absorbance values were recorded at 490 nm. Four parallels were measured for each data point.

### C4 deposition ELISA with diluted NHS on mannan- or AcBSA-coated surfaces

The assays were performed as in the case of C3 deposition from diluted NHS with the following modifications. (i) Plates were coated with 10 μg/ml mannan or 50 μg/ml AcBSA. (ii) The dilution of NHS was 60-fold. (iii) C4 deposition was measured using polyclonal rabbit anti-human C4c antibody (Q0369, DakoCytomation) as the primary antibody in a 2,000-fold dilution on a mannan-coated surface and in a 1,000-fold dilution on an AcBSA-coated surface. BSA acetylation was done as described (40).

### C4 deposition ELISA with pre-activated PRM:MASP complexes

The assay was performed as described previously (23, 38, 39). Greiner high-binding microtiter plates were coated with 1 mg/ml mannan in coating buffer. Wells were blocked with 20 mm HEPES, 140 mm NaCl, 5 mm EDTA, 0.1% Tween 20, pH 7.4, buffer (blocking buffer) for 30 min at room temperature. The plate was rinsed with 20 mm HEPES, 140 mm NaCl, 5 mm CaCl_2_, 0.1% Tween 20, pH 7.4, buffer (washing buffer). NHS was mixed with 40 mm HEPES, 2 m NaCl, 10 mm CaCl_2_, 0.1% Tween 20, pH 7.4, buffer in a 1:1 volume ratio and transferred onto the plate, which was incubated for 1 h at 37 °C. The plate was rinsed with 20 mm HEPES, pH 7.4, 1 m NaCl, 5 mm CaCl_2_, 0.1% Tween 20 buffer and then with washing buffer. Serial dilutions of the inhibitors were made in serum dilution buffer containing 1 μg/ml C4 α-chain. The samples were applied to the plate and incubated for 1 h at 37 °C. The plate was rinsed with blocking buffer, and polyclonal rabbit anti-human C4c antibody diluted 1,000-fold in blocking buffer was added to the wells, and the plate was incubated for 1 h at 37 °C. After washing with blocking buffer, horseradish peroxidase–conjugated monoclonal mouse anti-rabbit IgG antibody diluted 40,000-fold in blocking buffer was added, and the plate was incubated for 30 min at 37 °C. The plate was rinsed with washing buffer, and 1 mg/ml *o*-phenylenediamine dihydrochloride in 50 mm citrate, 0.1% H_2_O_2_, pH 5.0, buffer was added to the wells. After signal development (3–5 min), the reaction was stopped by adding 50 μl/well 1 m H_2_SO_4_, and the absorbance values were recorded at 490 nm. Four parallels were measured for each data point.

### Pathway-selective complement ELISAs using 2-fold diluted serum

The assays were carried out based on the work of Héja *et al.* (25).

### Lectin pathway

Microtiter plates were coated with 10 μg/ml mannan in coating buffer overnight at 4 °C. The wells were blocked with TBS/BSA/T. NHS was mixed in a 1:1 ratio with serial dilutions of the inhibitors made in 2-fold concentrate serum dilution buffer. The samples contained SPS at 100 μg/ml final concentration (40, 41). The serum:inhibitor samples were incubated for 30 min at room temperature. The plate was rinsed with TBS/Ca/T, and the samples were applied to the plate, which was incubated for 30 min at 37 °C. C4 deposition was measured as described above using polyclonal rabbit anti-human C4c antibody in a 5,000-fold dilution. Four parallels were measured for each data point.

### Classical pathway

The assays were performed as in the case of the LP measurement with the following modifications. (i) Plates were coated with 10 μg/ml human IgG. (ii) SPS was omitted from the buffers. Two parallels were measured for each data point.

### Alternative pathway

The assays were performed as in the case of the LP measurements with the following modifications. (i) Plates were coated with 10 μg/ml *Salmonella* lipopolysaccharide. (ii) Serum dilution buffer lacked CaCl_2_ and was supplemented with 10 mm EGTA. (iii) SPS was omitted from the buffers. (iv) C3 deposition was measured using polyclonal rabbit anti-human C3c antibody (A0062, DakoCytomation) in a 5,000-fold dilution. Two parallels were measured for each data point.

### Complement ELISAs with rat serum

Individual sera of Wistar rats were used in these experiments.

### C3 deposition ELISA with diluted rat serum

The assay was performed similarly to the C3 deposition assay with diluted NHS, but rat serum was used in a 70-fold dilution. The polyclonal rabbit anti-human C3c antibody recognizes the deposited rat C3 fragments, and it was used as primary antibody in a 2,000-fold dilution.

### C4 deposition ELISA with diluted rat serum

The assay was performed on mannan-coated ELISA plates similarly to the C4 deposition assay with diluted NHS, but rat serum was used in a 60-fold dilution. The polyclonal rabbit anti-human C4c antibody recognizes the deposited rat C4 fragments, and it was used as primary antibody in a 2,000-fold dilution.

### C5b-9 deposition ELISA with diluted rat serum

The assay was performed similarly to the C3 deposition assay with diluted NHS, but rat serum was used in a 50-fold dilution. Monoclonal mouse anti-rat C5b-9 antibody (sc-66190, Santa Cruz Biotechnology, Inc.) was used as primary antibody in a 1,000-fold dilution, and peroxidase-conjugated anti-mouse polyclonal antibody (AP308P, Merck) was used as secondary antibody in a 3,000-fold dilution.

### Blood coagulation assays

The effect of TFMI-2 variants on blood coagulation was tested in three standard assays: the TT, testing any direct effects on thrombin; the PT, testing any effects on the extrinsic pathway; and the APTT, testing any effects on the intrinsic pathway. Blood was collected from a healthy individual by vein puncture after informed consent. The blood was treated with sodium citrate (3.8%, w/v) and centrifuged. All three assays were performed on an automated CA-1500 instrument (Sysmex) with Innovin reagent (Dale Behring, Marburg, Germany).

## Author contributions

D. S., A. K., and R. S. investigation; D. S., A. K., and R. S. methodology; D. S. writing-original draft; D. S., A. K., R. S., P. G., and G. P. writing-review and editing; P. G. and G. P. conceptualization; P. G. and G. P. funding acquisition; G. P. resources; G. P. supervision.

## Supplementary Material

Supporting Information

## Footnotes

2 The abbreviations used are:

CS: complement system
AP: alternative pathway
CP: classical pathway
IRI: ischemia-reperfusion injury
LP: lectin pathway
MBL: mannan-binding lectin
MASP: MBL-associated serine proteinase
TFPI1: tissue factor pathway inhibitor
TFPI1 D2: the second Kunitz domain of TFPI1
TFMI: TFPI1-based MASP inhibitor
PRM: pattern recognition molecule
SFTI: sunflower trypsin inhibitor
NHS: normal human serum
AcBSA: acetylated BSA
SPS: sodium polyanethole sulfonate
TT: thrombin time
PT: prothrombin time
APTT: activated partial thromboplastin time
TBS: Tris-buffered saline.
