# Supplementary material for: Novel MASP-2 inhibitors developed via directed evolution of human TFPI1 are potent lectin pathway inhibitors
Source: J Biol Chem. 2019 Apr 5;294(20):8227–37. doi: 10.1074/jbc.RA119.008315 (PMC6527154; doi:10.1074/jbc.RA119.008315)
Supplement: Supporting Information [file supp_294_20_8227__index.html]

Novel MASP-2 inhibitors developed via directed evolution of human TFPI1 are potent lectin pathway inhibitors — TFPI1-based inhibitors inhibit both human and rat MASP-2 — Novel MASP-2 inhibitors developed via directed evolution of human TFPI1 are potent lectin pathway inhibitors — TFPI1-based inhibitors inhibit both human and rat MASP-2 — Supporting Information 

# Novel MASP-2 inhibitors developed via directed evolution of human TFPI1 are potent lectin pathway inhibitors

## Supporting Information

- Szak&#x00E1;cs\_et\_al\_Supporting Information (to be published online) - Technical details and supporting information.
