# Supplementary material for: Novel MASP-2 inhibitors developed via directed evolution of human TFPI1 are potent lectin pathway inhibitors
Source: J Biol Chem. 2019 Apr 5;294(20):8227–37. doi: 10.1074/jbc.RA119.008315 (PMC6527154; doi:10.1074/jbc.RA119.008315)
Supplement: Supporting Information [file supp_RA119.008315_144142_1_supp_307734_pp32gv.pdf]

Supporting information

Figure S1.

pTac promoter

GTTGACAATTAATCATCGGCTCGTATAATGTGTGGAATTGTGAGCGGATAACAATTTCACACAGGAAACAG

pTac promoter MalE signal

CCAGTCCGTTTAGGTGTTTTCACGAGCACTTCACCAACAAGGACCATAG**CAT** **ATG**AAAATAAAAACAGGT

M K I K T G

MalE signal Flag-tag

GCACGCATCCTCGCATTATCCGCATTAACGACGATGATGTTTTCCGCCTCGGC**GCTAGC**ATCG GATTATA

A R I L A L S A L T T M M F S A S A L A S D Y K

Flag-tag Ser/Gly linker TFPI-D2

AAGACGATGATGACA**AA** **GCTT**CCGGCGGG**TCCGGA**GGCTCGGGC AAACCGGACTTCTGCTTCCTGGAAG

D D D D K A S G G S G G S G K P D F C F L E E

TFPI-D2

AAGACCCGGGTATCTGCCGTGGTTACATCACCCGTTACTTCTACAACAACCAGACCAAACAGTGCGAACGT

D P G I C R G Y I T R Y F Y N N Q T K Q C E R

P3 P1 P1’P2’P3’P4’

TFPI-D2

TTCAAATACGGTGGTTGCCTGGGTAACATGAACAACTTCGAAACCCTGGAAGAATGCAAAAACATCTGCGA

F K Y G G C L G N M N N F E T L E E C K N I C E

TFPI-D2 Ser/Gly linker p8 coat protein

AGACGGT GGCGGCAGCGGCGGCAGCGGCGG**GAGCTC**CAGC GCCGAGGGTGACGATCCCGCAAAAGCGGC

D G G G S G G S G G S S S A E G D D P A K A A

p8 coat protein

CTTTAACTCCCTGCAAGCCTCAGCGACCGAATATATCGGTTATGCGTGGGCGATGGTTGTTGTCATTGTCG

F N S L Q A S A T E Y I G Y A W A M V V V I V G

p8 coat protein

GCGCAACTATCGGTATCAAGCTGTTTAAGAAATTCACCTCGAAAGCAAGCTAA**TCTAGA**

A T I G I K L F K K F T S K A S *

Figure S1. - DNA and amino acid sequence of the TFPI1 D2 – p8 fusion gene
Restriction endonuclease cleavage sites enabling easy replacement of the individual functional modules are shown in bold. The functional modules of the sequence are underlined and indicated above the sequence. Randomized positions between P3-P4’ of the TFPI1 D2 proteinase-binding loop are highlighted with grey. The P2 Cys forms a structurally indispensable disulfide therefore it was kept unchanged.

Figure S2

```
             His6-tag                             S100A4
CCATGGGC CATCACCATCACCATCAC GCTAGC ATGGCGTCCCCTCTGGAGAAGGCCCTGGATGTGATG
  M  G   H  H  H  H  H  H   A  S   M  A  S  P  L  E  K  A  L  D  V  M

                                 S100A4
GTGTCCACCTTCCACAAGTACTCGGGCAAAGAGGGTGACAAGTTCAAGCTCAACAAGTCAGAACTAAAGGA
V  S  T  F  H  K  Y  S  G  K  E  G  D  K  F  K  L  N  K  S  E  L  K  E

                                 S100A4
GCTGCTGACCCGGGAGCTGCCCAGCTTCTTGGGGAAAAGGACAGATGAAGCTGCTTTCCAGAAGCTGATGA
 L  L  T  R  E  L  P  S  F  L  G  K  R  T  D  E  A  A  F  Q  K  L  M  S

                                 S100A4
GCAACTTGGACAGCAACAGGGACAACGAGGTGGACTTCCAAGAGTACTGTGTCTTCCTGTCCTCCATCGC
  N  L  D  S  N  R  D  N  E  V  D  F  Q  E  Y  C  V  F  L  S  S  I  A

                     S100A4                                        TEV-site
CATGATGTCTAACGAATTCTTTGAAGGCTTCCCAGATAAGCAGCCCAGGAAGAAA GTCGAC GACTACGA
 M  M  S  N  E  F  F  E  G  F  P  D  K  Q  P  R  K  K   V  D   D  Y  D

     TEV protease cleavage site                   TFPI D2
CATCCCGACTACCGAAAACCTGTACTTCCAGGGATCC AAACCGGACTTCTGCTTCCTGGAAGAAGACCCG
 I  P  T  T  E  N  L  Y  F  Q  G  S   K  P  D  F  C  F  L  E  E  D  P

                                TFPI D2
GGTATCTGCCGTGGTTACATCACCCGTTACTTCTACAACAACCAGACCAAACAGTGCGAACGTTTCAAATA
G  I  C  R  G  Y  I  T  R  Y  F  Y  N  N  Q  T  K  Q  C  E  R  F  K  Y
   P3     P1 P1'P2'P3'P4'
                                TFPI D2
CGGTGGTTGCCTGGGTAACATGAACAACTTCGAAACCCTGGAAGAATGCAAAAACATCTGCGAAGACGGTT
 G  G  C  L  G  N  M  N  N  F  E  T  L  E  E  C  K  N  I  C  E  D  G  *

TAATAAGCTTGGCACTCGAG
```

Figure S2. - DNA and amino acid sequence of the S100A4 – TFPI1 D2 fusion gene
Restriction endonuclease cleavage sites enabling easy replacement of the individual functional modules are shown in bold. The functional modules of the sequence are underlined and indicated above the sequence. Positions between P3-P4' of the TFPI1 D2 proteinase-binding loop that were allowed to evolve in the phage display process are highlighted with grey.

Table S1. - Sequences of the primers and synthetic genes used in the study

The function of oligonucleotides and synthetic gene constructs	The sequence of DNA primers and synthetic genes
The TFPI1 D2 synthetic gene.	***TCCGGA****GGCTCGGGC*AAACCGGACTTCTGCTTCCTGGAAGA<br>AGACCCGGGTATCTGCCGTGGTTACATCACCCGTTACTTCTA<br>CAACAACCAGACCAAACAGTGCGAACGTTTCAAATACGGTG<br>GTTGCCTGGGTAACATGAACAACTTCGAAACCCTGGAAGAA<br>TGCAAAAACATCTGCGAAGACGGT*GGCGGCAGCGGCGGCAG*<br>*CGGCGG***GAGCTC***
The TFPI1 D2 P3-P4' STOP primer.	CCTGGAAGAAGACCCGGGTTAATGCTAATAATAATAATAAC<br>GTTACTTCTACAACAACCAGACC
The TFPI1 D2 P3-P4' library primer.	CCTGGAAGAAGACCCGGGTNNKTGCNNKNNKNNKNNKNNK<br>CGTTACTTCTACAACAACCAGACC
The TFPI BamHI forward primer used to clone TFPI1 D2 into the pMal-p2G plasmid.	AGTA**GGATCC**AAACCGGACTTCTGC
The TFPI HindIII reverse primer used to clone TFPI1 D2 into the pMal-p2G plasmid.	GTAT**AAGCTT**ATTAACCGTCTTCGCAGATG
The TFMI-2a mutagenesis primer.	CCTGGAAGAAGACCCGGGTATCTGCAAACTGTTCTTCATCCG<br>TTACTTCTACAACAACCAGACC
The TFMI-2b mutagenesis primer.	CCTGGAAGAAGACCCGGGTCCGTGCCGTGCGGTGAAACGTC<br>GTTACTTCTACAACAACCAGACC
The TFMI-2c mutagenesis primer.	CGGGTGTGTGCCGTGCGGTGAAACG
The reverse primer used in the first PCR of megaprimer PCR mutagenesis.	CTAGT**CTCGAG**TGCCAAGC
The forward primer used in the second PCR of megaprimer PCR mutagenesis.	GATGTCCGCTTTCTGGTATGC

The restriction endonuclease sites are shown in bold. Bases introducing mutations in mutagenesis reactions are underlined. Sequences encoding for Ser/Gly linkers, His_6-tag or TEV proteinase cleavage site are shown in italic.

Table S2. - Amino acid and DNA sequences of the 43 unique human MASP-2 binding clones

	Amino acid sequence							DNA sequence
	P3	P2	P1	P1'	P2'	P3'	P4'	
1	L	C	R	A	L	M	K	TTGTGCCGTGCTCTGATGAAG
2	Y	C	R	A	H	K	E	TATTGCAGGGCTCATAAGGAG
3	P	C	R	A	A	K	R	CCGTGCCGGGCGGCGAAGCGG
4	Y	C	R	G	V	R	I	TATTGCCGTGGGGTTAGGATT
5	L	C	R	A	L	S	M	CTGTGCCGTGCTCTGTCTATG
6	M	C	R	A	L	I	S	ATGTGCCGTGCTTTGATTAGT
7	V	C	R	S	A	R	R	GTGTGCAGGTCGGCGAGGAGG
8	F	C	R	A	I	Q	Q	TTTTGCCGTGCGATTCAGCAG
9	Y	C	R	A	I	Q	M	TATTGCCGGGCGATTCAGATG
10	P	C	R	A	L	K	V	CCTTGCCGTGCTTTGAAGGTG
11	W	C	R	A	I	I	K	TGGTGCAGGGCTATTATTAAG
12	F	C	R	A	V	Q	L	TTTTGCCGTGCGGTGCAGCTG
13	F	C	R	A	L	N	I	TTTTGCAGGGCTCTTAATATT
14	P	C	R	A	L	I	R	CCTTGCCGTGCGTTGATTCGT
15	M	C	R	A	V	K	R	ATGTGCCGTGCGGTGAAGAGG
16	F	C	R	A	V	K	F	TTTTGCAGGGCTGTTAAGTTT
17	L	C	R	G	A	K	R	CTGTGCAGGGGTGCTAAGAGG
18	Y	C	R	A	V	V	F	TATTGCCGTGCGGTTGTTTTT
19	Y	C	R	A	M	K	V	TATTGCCGTGCGATGAAGGTT
20	Y	C	R	A	V	R	V	TATTGCCGTGCGGTTAGGGTT
21	A	C	R	A	V	K	R	GCGTGCAGGGCGGTTAAGAGG
22	P	C	R	A	S	S	V	CCTTGCCGGGCTTCTTCGGTG
23	F	C	R	T	A	H	V	TTTTGCCGGACGGCTCATGTG
24	L	C	R	A	L	L	H	TTGTGCCGGGCTCTGCTTCAT
25	P	C	R	A	V	R	K	CCTTGCCGGGCGGTTCGGAAG
26	L	C	R	A	V	S	F	CTGTGCAGGGCTGTTTCCTTT
27	F	C	R	S	L	S	Y	TTTTGCCGGTCTCTTAGTTAT
28	L	C	R	A	L	K	I	CTTTGCAGGGCGTTGAAGATT
29	V	C	R	S	A	I	R	GTGTGCAGGTCGGCTATTCGG
30	T	C	L	S	D	F	N	ACTTGCTTGAGTGATTTTAAT
31	F	C	R	G	L	K	V	TTTTGCCGTGGTTTGAAGGTT
32	L	C	R	T	A	K	V	CTTTGCCGGACGGCTAAGGTT
33	F	C	R	A	L	H	L	TTTTGCAGGGCTCTGCATTTG
34	Q	C	R	A	V	R	R	CAGTGCAGGGCGGTTAGGAGG
35	P	C	R	A	V	S	R	CCTTGCAGGGCTGTTTCGCGG
36	Q	C	R	A	M	R	R	CAGTGCAGGGCTATGCGTCGG

37	F	C	R	A	L	R	S	TTTTGCAGGGCGTTGCGTAGT
38	L	C	R	A	V	K	V	CTGTGCCGTGCGGTGAAGGTT
39	F	C	R	A	I	K	D	TTTTGCCGTGCTATTAAGGAT
40	L	C	R	A	A	L	R	CTGTGCAGGGCGGCGTTGCGG
41	L	C	R	A	V	K	L	CTGTGCCGGGCTGTGAAGTTG
42	Q	C	R	G	A	R	Q	CAGTGCCGTGGGGCGAGGCAG
43	P	C	R	G	V	R	R	CCGTGCCGTGGTGTGCGGCGG

Table S3. - Amino acid and DNA sequences of the 53 unique rat MASP-2 binding clones

	Amino acid sequence							DNA sequence
	P3	P2	P1	P1'	P2'	P3'	P4'	
1	P	C	R	A	L	G	G	CCGTGCAGGGCGCTGGGGGGG
2	V	C	R	A	M	A	S	GTTTGCCGGGCGATGGCGTCT
3	V	C	R	G	Y	A	L	GTTTGCCGTGGGTATGCTTTG
4	V	C	R	G	L	K	A	GTGTGCCGGGGGTTGAAGGCT
5	V	C	R	G	Y	A	E	GTTTGCAGGGGTTATGCTGAG
6	V	C	R	A	W	F	I	GTTTGCCGTGCTTGGTTTATT
7	P	C	R	A	A	T	E	CCGTGCCGGGCGGCTACGGAG
8	P	C	R	A	L	A	Y	CCTTGCAGGGCTTTGGCGTAT
9	V	C	R	A	L	E	W	GTTTGCAGGGCTTTGGAGTGG
10	P	C	R	A	L	A	G	CCGTGCCGGGCGTTGGCTGGG
11	V	C	R	A	I	T	I	GTTTGCAGGGCTATTACGATT
12	P	C	R	S	L	I	N	CCGTGCCGGTCGTTGATTAAT
13	P	C	R	A	L	R	V	CCGTGCCGGGCTTTGCGGGTT
14	V	C	R	G	L	A	E	GTGTGCCGGGGGCTGGCGGAG
15	V	C	R	A	L	R	V	GTTTGCCGGGCGCTTCGGGTT
16	P	C	R	A	I	N	Y	CCGTGCCGGGCTATTAATTAT
17	V	C	R	S	L	Q	F	GTGTGCAGGTCGTTGCAGTTT
18	P	C	R	A	L	L	H	CCGTGCCGGGCTTTGTTGCAT
19	V	C	R	A	L	L	V	GTTTGCCGTGCTTTGTTGGTG
20	V	C	R	G	Y	A	S	GTTTGCCGTGGGTATGCTAGT
21	P	C	R	S	L	T	Y	CCTTGCCGTTCGCTTACTTAT
22	P	C	R	A	L	F	E	CCGTGCCGGGCTCTGTTTGAG
23	I	C	R	A	L	S	L	ATTTGCCGGGCTCTGTCCCTT
24	P	C	R	A	L	I	E	CCGTGCAGGGCTCTGATTGAG
25	G	C	R	A	L	S	A	GGGTGCAGGGCTTTGTCGGCT
26	V	C	R	S	L	N	L	GTTTGCAGGTCTCTGAATTTG
27	P	C	R	A	Y	L	Q	CCTTGCCGTGCTTATCTCCAG
28	V	C	R	A	L	A	Y	GTTTGCCGTGCTCTTGCGTAT
29	V	C	R	A	V	V	E	GTGTGCCGGGCGGTGGTTGAG
30	I	C	R	A	L	I	R	ATTTGCCGGGCGCTTATTCGG
31	V	C	R	A	L	I	L	GTTTGCCGTGCGCTTATTTTG
32	V	C	R	A	L	V	L	GTTTGCAGGGCTCTGGTTCTT
33	V	C	R	A	L	I	T	GTGTGCCGTGCTCTGATTACT
34	V	C	R	A	L	F	L	GTGTGCAGGGCTCTTTTTCTT
35	P	C	R	A	L	T	V	CCTTGCAGGGCGCTGACTGTT
36	V	C	R	G	L	L	K	GTTTGCAGGGGGCTGCTGAAG

37	P	C	R	A	L	R	Q	CCTTGCAGGGCTTTGCGGCAG
38	I	C	R	A	L	Q	M	ATTTGCCGTGCGTTGCAGATG
39	P	C	R	A	L	Y	L	CCTTGCCGTGCTCTGTATTTG
40	V	C	R	A	L	I	N	GTGTGCAGGGCTCTTATTAAT
41	P	C	R	S	L	I	F	CCGTGCCGGTCGTTGATTTTT
42	P	C	R	G	L	A	R	CCGTGCAGGGGGCTTGCTAGG
43	V	C	R	A	V	L	Y	GTTTGCAGGGCGGTTCTTTAT
44	V	C	R	A	L	T	V	GTGTGCAGGGCGTTGACTGTT
45	P	C	R	A	M	T	L	CCGTGCCGGGCTATGACGTTG
46	I	C	R	G	L	I	L	ATTTGCCGTGGTCTGATTTTG
47	P	C	R	G	L	K	S	CCGTGCAGGGGGCTTAAGAGT
48	V	C	R	S	L	A	L	GTTTGCCGTTCTTTGGCTCTG
49	P	C	R	S	L	K	M	CCGTGCCGGTCGCTTAAGATG
50	V	C	R	A	C	A	Y	GTTTGCAGGGCGTGTGCGTAT
51	P	C	R	G	L	S	C	CCTTGCCGGGGTCTTTCGTGT
52	V	C	R	S	C	S	I	GTTTGCCGGTCTTGTTCTATT
53	P	C	R	S	L	I	C	CCTTGCCGTAGTCTGATTTGT
